# “Just because you’re pregnant, doesn’t mean you’re sick!” A qualitative study of beliefs regarding physical activity in black South African women

**DOI:** 10.1186/s12884-016-0963-3

**Published:** 2016-07-19

**Authors:** Estelle D. Watson, Shane A. Norris, Catherine E. Draper, Rachel A. Jones, Mireille N. M. van Poppel, Lisa K. Micklesfield

**Affiliations:** Centre for Exercise Science and Sports Medicine, School of Therapeutic Sciences, Faculty of Health Sciences, University of Witwatersrand, Johannesburg, South Africa; MRC/WITS Developmental Pathways for Health Research Unit, Department of Paediatrics, School of Clinical Medicine, Faculty of Health Sciences, University of Witwatersrand, Johannesburg, South Africa; Department of Human Biology, Faculty of Health Sciences, Division of Exercise Science and Sports Medicine, University of Cape Town, Cape Town, South Africa; Early Start Research Institute, Faculty of Social Sciences, University of Wollongong, Wollongong, Australia; Institute of Sport Science, University of Graz, Graz, Austria; Department of Public and Occupational Health, EMGO Institute for Health and Care Research, VU University medical center, Amsterdam, The Netherlands

**Keywords:** Pregnancy, Low income, African, Physical activity, Exercise, Beliefs, Barriers

## Abstract

**Background:**

Despite the benefits of physical activity during pregnancy, the physiological and psychological changes that occur during this unique period may put women at greater risk of being sedentary. Lifestyle and environmental transitions have left black South African women at increased risk of physical inactivity and associated health risks. Therefore, the aim of this qualitative study was to describe the beliefs regarding physical activity during pregnancy in an urban African population.

**Methods:**

Semi-structured interviews (*n* = 13) were conducted with pregnant black African women during their third trimester. Deductive thematic analysis was completed based on the Theory of Planned Behaviour. Coding and analysis was completed with the assistance of ATLAS.ti software.

**Results:**

Participants had a mean age of 28 (19–41) years, and a mean BMI of 30 (19.6–39.0) kg/m^2^. Although the majority of women believed that physical activity was beneficial, this did not appear to translate into behaviour. Reported reasons for this included barriers such as pregnancy-related discomforts, lack of time, money and physical activity related education, all of which can contribute to a reduced perceived control to become active. Opportunities to participate in group exercise classes was a commonly reported facilitator for becoming active. In addition, influential role players, such as family, friends and healthcare providers, as well as cultural beliefs, reportedly provided the women with vague, conflicting and often discouraging advice about physical activity during pregnancy.

**Conclusions:**

This study provides new theoretical insight on the beliefs of urban South African pregnant women regarding physical activity. Findings from this study suggest a holistic approach to improve physical activity compliance during pregnancy, inclusive of physical activity education and exercise opportunities within a community setting.

This study presents critical formative work upon which contextually and culturally sensitive interventions can be developed.

## Background

Pregnancy is a unique time in a woman and her unborn childs’ life course, and thus it is unsurprising that maternal health is a worldwide health priority [1]. Although much of the focus of healthcare has been on reducing the direct causes of maternal morbidity, recent years have seen a shift towards addressing modifiable health risk factors, such as diet and physical activity. Indeed, pregnancy may be a critically receptive period to improve the health outcomes for both the mother and her unborn child [2, 3].

The benefits of an active pregnancy are well documented and studies have demonstrated a reduced risk of pre-eclampsia, gestational diabetes mellitus (GDM) and gestational hypertension [4]. Meeting the recommended 150 min of moderate physical activity per week [5] during pregnancy may also lead to improved course of delivery and provide a protective effect against low birth weight and prematurity [6]. Furthermore, it may play a crucial role in preventing maternal obesity, excessive gestational weight gain and infant obesity [2]. This may be particularly important for women from low- and middle- income countries (LMICs), where low socio-economic status (SES) in a transitioning environment, has been reported as a risk factor for high prevalence of obesity, physical inactivity, impaired glucose intolerance and GDM [7–10].

However, despite these recommendations, pregnant women have been shown to be less active than their non-pregnant counterparts, and tend to decrease their activity levels during pregnancy [11]. Previous studies from Brazil indicate that pregnant women are susceptible to low levels of physical activity [12], despite their knowledge of the benefits [13]. Pregnancy-specific psychological factors such as fear of miscarriage, and physical factors such as fatigue and increased stomach size, may contribute to a reduction in activity levels. These combined psychological and physiological factors may make pregnancy a vulnerable period for reducing physical activity levels and increasing sedentary behaviours [14].

Understanding the social, cognitive and behavioural factors that predict and describe physical activity participation becomes essential for understanding and intervening in this potentially vulnerable population group. To this end, the Theory of Planned Behaviour (TPB) is a useful approach for understanding, explaining and predicting physical activity behaviours [15, 16], including during pregnancy [17, 18]. It involves three main domains, namely, behavioural beliefs/attitudes (the perceptions of the consequences of the behaviour), perceived behavioural control (PBC)/control beliefs (the perceived degree of control over performance of the behaviour) and subjective norms/normative beliefs (the social pressure, or perceptions of what significant others think about the behaviour) [17, 19]. In order for researchers to plan interventions or change public health policy, the multidimensional determinants of physical activity participation need to be understood, and TPB provides a valuable framework in which to do this.

There is currently little research that has investigated South African women’s beliefs or factors that influence their physical activity behaviours. Therefore, the aim of this study is to determine the beliefs of black South African women regarding physical activity during pregnancy, and therefore provide formative theory on which to design effective physical activity interventions during pregnancy.

## Methods

### Study sample

Pregnant (29–33 weeks gestation) participants were recruited from the MRC/Wits Developmental Pathways for Health Research Unit (DPHRU), based at Chris Hani Baragwaneth Hospital (CHBH), Soweto, South Africa. Soweto, an English abbreviation for South Western Township, is one of the largest urban areas in South Africa, with over one million inhabitants. Although the majority of its inhabitants come from low-income households, it also has a rising middle class and this, combined with historical apartheid-related poverty, makes it a complex mix for social inequalities and health care issues (www.soweto.co.za). 

A purposive sampling approach was used to include a homogenous group of singleton, black pregnant women with non-complicated pregnancies in the third trimester [20]. Women were purposively recruited from a public antenatal hospital, as this is a proxy for low to middle socioeconomic status in South Africa. A sample matrix [20] was designed to include a sample size of 15 women; however, data saturation was reached after 13 interviews. The women attended the DPHRU for non-clinical, research purposes every 4–6 weeks during their pregnancy as part of a larger study (Soweto First 1000 Days study). Reporting of methods has been based on the Relevance, Appropriate, Transparency and Soundness (RATS) review guidelines to ensure rigour and quality [21, 22].

### Theoretical framework

This study adopted a deductive logic approach [20] and was grounded in the Theory of Planned Behaviour (TPB) [16]. Since this research was aimed at eliciting personal experiences and perceptions regarding the concepts of physical activity in its broader sense, an interpretative phenomenological analysis approach was adopted [20]. Within the construct of PBC, the current study did not explicitly address the areas of self-efficacy or controllability. Instead, this was measured indirectly based on beliefs regarding the perceived ease or difficulty in participating in and performing a behaviour (e.g. I don’t have the money to attend the gym) [16, 18, 19, 23]. This belief based method of measuring PBC may have the advantage of providing underlying cognitive insights that would otherwise be missed if measured directly [19]. The rationale, as described by Ajzen [16], is that the more resources and opportunities available, and the less or more manageable the barriers, the increased confidence to participate in the behaviour, and the higher the degree of perceived behavioural control exhibited [19]. Furthermore, perceived control over the performance of a behaviour also involves various intrinsic and extrinsic factors that may serve to help or hinder the behaviour. Although it may be argued that self-efficacy is measured through intrinsic factors, and controllability through external factors, these concepts may reflect both beliefs and were therefore not treated as mutually exclusive in the current study [19]. Since self-efficacy and controllability are also found to be closely related, both these constructs can be seen as influencers of an individual’s perceived ability to control their behaviour [19].

### Data collection

Semi-structured interviews were conducted between March – December 2014. All interviews were conducted in English, which is the language common to most inhabitants of Soweto. Each interview was conducted at the DPHRU by the researcher (EW), who did not have a clinical role in the participants’ visits to DPHRU. Open-ended questions, with probes, were developed using the TPB theoretical framework, and informed by previous studies [24–27]. They were designed to facilitate breadth and depth of conversation and provide information-rich discussion [20]. Although the concept of perceived behavioural control was not explicitly asked in the interview guide, questions related to factors that obstruct or facilitate participation in physical activity were used to elicit the participants’ perception about their control over the achievement of the behaviour [24]. The guide was reviewed for qualitative properties by an expert in the field of public health, and pilot tested prior to data collection. Each interview, which lasted between 45 and 80 min, was audio-recorded and notes on non-verbal actions were taken by the researcher.

Demographic and pregnancy medical history information was collected on all participants using an interview-administered questionnaire. Furthermore, physical activity levels were measured using the Global Physical Activity Questionnaire (GPAQ), a valid and reliable tool that measures physical activity in various domains, including work, travel and recreation [28].

### Data analysis

Recorded data were transcribed verbatim and reviewed by the researcher for accuracy. A formal thematic analysis process was undertaken, including familiarisation, construction of an initial framework within the TPB, indexing and sorting, reviewing, data summary, constructing categories and identifying links and patterns [20]. A data analysis software tool (ATLAS.ti 5.0) was used to organise the codes and themes into a structure for analysis. Major themes are represented according to frequency, and thematic saturation was reached by the ninth interview. To ensure reliability of the coding framework the initial thematic coding was completed and left, and then done again one month later. In addition, a sample of three transcripts were peer-reviewed to agree on the coding framework.

## Results

Participants (*n* = 13) ranged in age from 19 to 41 years of age (Table 1). The average BMI was 30 kg/m^2^ (range: 19.6–39.0 kg/m^2^). Majority of the women (69 %) were single, and 59 % had only a secondary education level. Over half (54 %) of the women were classified as inactive according to the GPAQ cut-off of less than 600 MET-minutes per week requirements [28].

**Table 1:** A summary of participant characteristics (*n* = 13)

	Mean (range) / %
Age in years	28 (19–41)
Baseline (<14 weeks gestation) BMI (kg/m^2^)	30 (19.6–39.0)
Marital status	
Single	69
Married	31
Education	
Secondary school	59
Professional/technical training	8
University	33
Employment	
Skilled manual labour	25
Unskilled manual labour	17
Clerical/administrative/student	33
Unemployed	25
HIV status	
Positive	33
Negative	67
Household inventory (/9)	
< 6	17
6–7	83
> 8	0
Paternal age in years	34 (23–44)
GPAQ physical activity status	
Active	46
Inactive	54

The qualitative findings of the study are presented according to the following typologies (i) behavioural beliefs and attitudes; (ii) perceived behavioural control (PBC) and (iii) subjective norms/normative beliefs. Common views and experiences of participants are presented as verbatim quotes, and described using the participant’s BMI and physical activity status (according to the GPAQ).

### Behavioural beliefs and attitudes

Although some women warned against the potential adverse effects of alcohol, drugs, stress and heavy work during pregnancy, the majority of participants were aware of the importance of adopting healthy behaviours during pregnancy including a healthy diet, exercise, and sufficient rest.

*“A good diet. Exercise, but not excessive exercise. Good rest is also very important.” (BMI 30 kg/m*^*2*^*; Active)*

All the women who participated in the interviews believed that physical activity was in some way beneficial. For most of the women, walking appeared to be the preferred form of physical activity. The most popular perceived benefits reported by participants included the belief that physical activity would help to prepare the body for labour, reduce labour time, and that the baby would also be prepared:

“*It helps relax the muscles so that when you give birth, the muscles are not tight, they’re used to expanding, yes, that movement and even the baby is used to the movement and he won’t be surprised…it prepares the body and the baby also.” (BMI 24.6 kg/m*^*2*^*; Active)*

One woman explained:

*“I think for my own health…Apart from the birth, I think for myself, my own health, my heart, and I also feel the skin, I don’t know, when I’m walking, I feel like my skin is breathing more. So I think it keeps it healthier, you know? The more it sweats and, I think it’s good….It helps you relax as well. When you [walk], it is kind of therapeutic for me, I don’t know.” (BMI 30 kg/m*^*2*^*; Active)*

For many of the women, physical activity was defined as an activity of daily living, such as occupational and household tasks, and few described it as recreational. Throughout the interviews, many women presented the idea that being busy was synonymous with being active:

“*If you have a job, that job keeps you busy, you are always busy so the baby is busy too, but if you are always sleeping the baby will be sleeping too.” (BMI 32 kg/m*^*2*^*; Active)*

Another woman commented:

*“Physical activity, I think, is just keeping active…when you’re home walking around and not sitting too still. I have a baby so she keeps me very busy. If you have a child you don’t have a problem with physical activity because you’re always running after them.” (BMI 30 kg/m*^*2*^*; Active)*

While participants reported on the benefits of physical activity, several women were also conscious of ‘overdoing’ it during pregnancy. Certain occupational, household and recreational tasks were described as too vigorous and were therefore perceived as unsafe. Many women revealed a general uncertainty, without always knowing why these activities were deemed unsafe. The perception as to whether a certain activity was safe or not, often originated from advice from family and friends, or listening to their own bodies:

“*I can talk about me…we used to go and fetch water at the tap, we must use the bucket and …when I pick it up, I can feel something inside me that is telling me that this thing is heavy just leave it, you know.” (BMI 30.6 kg/m*^*2*^*; Active)*

One woman commented:

*“I don’t know about swimming…you know you’re working on all muscles of your body, so I don’t know what impact that would have. Things like bending over, squatting… I always feel because it’s very difficult… I think maybe it’s hurting the baby.” (BMI 30 kg/m*^*2*^*; Active)*

### Perceived behavioural control beliefs

The women in the current study discussed various barriers, or obstacles, to participating in physical activity, which may influence the perceived control that women have over their physical activity behaviour, therefore reducing the time spent being physically active. The most frequently reported intrinsic barriers included tiredness, morning sickness, interrupted sleep and discomfort due to the size of the stomach. Participants appeared to display a reduced self-efficacy, and described the increasing difficulty in performing physical activity due to the pregnancy-associated physical changes. This was combined with a limited perceived control over their activity levels, where the unborn baby was described as determining the amount of physical activity done:

*“You know, this person is heavy, that you’re carrying….you get very tired. And being tired makes you sit down more, and you’re not very… you can’t do much.” (BMI 26.8 kg/m*^*2*^*; Active)*

Another participant commented:

*“You’re tired quicker when you're pregnant. I can tell when I can’t take anymore, so I just stop when I feel like that’s enough. Pregnancy is quite interesting, because you have those bouts of activity, you know, being extremely active and you do not know where it comes from and then the next minute you’re, like, I don’t want to do anything. I’m tired. So, it’s quite interesting in that sense. It differs from woman to woman. It depends on how heavy your belly is.” (BMI 30 kg/m*^*2*^*; Active)*

On the other hand, one woman described her belief that these barriers are within her control, and was determined not to allow the pregnancy to affect her behaviour:

“*They just say that this pregnancy it’s too tiring…so if you let the pregnancy control you, you won’t do anything in life truly. It does not mean that the baby just took over your body or your everything, you must exercise, you must eat well and do everything just the way that you are, you are not sick you are just pregnant.” (BMI 32 kg/m*^*2*^*; Active)*

Commonly reported extrinsic barriers included limited time or money available for physical activity. Prioritising their resources to meet their family’s needs appeared to diminish the participants’ perception of control towards being active:

*“I don’t have the time [to go to the gym]. When I’m coming to work I’m tired seriously. When you are off you are supposed to do the things at home so there is not time to go to gym.” (BMI 37.4 kg/m*^*2*^*; Active)*

Another woman commented:

*“Some [people] do not have the money to buy healthy food. Yes it [gym] is expensive, and that money you can use for the child…to buy medication for that child or milk.” (BMI 32.2 kg/m*^*2*^*; Inactive)*

Another extrinsic barrier noted by many women was the lack of information provided by healthcare professionals about physical activity during pregnancy. Many women reported receiving little information at the antenatal clinics, and information that was provided was often vague or non-specific. Some women mentioned that, within the healthcare system, education regarding communicable diseases was prioritised over providing information on the benefits of physical activity. Added to this, many women reported bad experiences at their antenatal clinics that prevented them from asking any physical activity-related questions:

*“They [the nurses] are just teaching HIV and AIDS and that’s it. There’s no time to ask questions or anything, you know…” (BMI 24.6 kg/m*^*2*^*; Active)*

Another woman agreed:

“*No to be honest those people [the nurses] are mean and they’re not helpful. I don’t know, maybe they don’t like pregnant women, we are making their lives difficult somehow. They don’t seem to care that much so I wouldn’t trust someone who doesn’t care about you at all. No I’m very intimidated by them so I do not ask questions, I rather ask friends and family.” (BMI 29.4 kg/m*^*2*^*; Inactive)*

In fact, one women articulated the importance of being provided with correct information to improve her perceived behavioural control, noting that with advice and education she would be more likely to be active:

“*I don’t know, I’m just too lazy to do them [exercise], I don’t know. Or maybe I don’t know the importance of doing them.” (BMI 29.4 kg/m*^*2*^*; Inactive)*

Many women also discussed extrinsic and environmental influences that would assist them in becoming more active. They were particularly supportive of having community-based group exercise classes aimed at pregnant women. A supportive and safe environment appeared to be a motivator to become active, and may therefore improve their perceived behaviour control in this area. Many of the women felt that not only would these community facilities improve their physical activity behaviour, but that it would provide an opportunity to expand their social support network:

*“Having antenatal classes in my neighbourhood, that would be awesome actually. Because they focus more on pregnant woman so I’ll feel much safer. I know I can do anything that they tell me to do there; because I know it’s not going to harm the baby. That would be great…I just want my baby to be safe.” (BMI 29.4 kg/m*^*2*^*; Inactive)*

And another agreed:

*“Pregnant ladies coming together, doing such [exercises], it would help a lot. If I had a group like that next to me, I would have been doing it…because that’s where you get to know also the other experiences and what other ladies go through.” (BMI 26.8 kg/m*^*2*^*; Active)*

### Subjective norms/normative beliefs

Healthcare providers were reported by some of the women as potential social influencers of their physical activity behaviours. However, since they provided very little education, healthcare workers were unlikely to provide any social pressure when it comes to participating in physical activity:

*“If they [the gynaecologist] also gave us information on that, that would help – the exercise positions, show you that when you’re pregnant you can do this, or in late pregnancy, you can’t do this. They don’t tell you…they never even mentioned to me to do exercise.” (BMI 30 kg/m*^*2*^*; Active)*

Due to this lack of information, many women turned to family, friends or the media for advice regarding pregnancy. However, in most cases, this advice encouraged women to reduce their activity levels during pregnancy, as a time to “take it easy”. Furthermore, this advice to reduce activity levels was coupled with the fact that many women did not have family or friends that participated in, or talked about the importance of, physical activity:

*“Just because you’re pregnant it doesn’t mean you’re sick, you’re not sick at all…go ahead and do what you normally would do, your daily routines as usual, keep them going until you can …You know, it’s so easy to be spoiled. My family …they used to just spoil me, but no, don’t do this, don’t go down, don’t pick it up, don’t do this, you know? So you get spoiled easily, and it’s easy for you to go, ah, I don’t feel like doing this, and get so lazy…” (BMI 26.8 kg/m*^*2*^*; Active)*

One woman described her friend’s reactions to being active:

*“Most of my friends are not physically active. If I even mention walking, they’re like, "Why would you …? “Only dogs walk” you know? I’m like, "No, it’s good for you!" They always want to drive, so yes. That’s the experience that I’ve always had. I don’t know if it’s a South African thing, but most people just want to get into a car. My husband is, every time I say, let’s go on a walk he says no. He doesn’t feel a need. Whereas for me it is…kind of…a need.” (BMI 30 kg/m*^*2*^*; Active)*

Many of the participants mentioned cultural beliefs and practices during pregnancy that are passed down through the generations. Often the women noted that these beliefs and practices are followed without knowing the reasoning behind them. One woman described the beliefs passed on from her family:

“*We have a lot of indigenous knowledge on pregnancy and, you know, as African people, you’re taught that at a very young age. "Do this, don’t …" You know, some of them are silly, but…! Like, don’t stand by the door, you know? Okay, some are just common knowledge, it’s like common sense. Don’t stand for too long. Maybe that’s why they say don’t stand at the door, but they have their own reasons, like superstitious reasons. You know, sometimes I remember things my mom used to tell me not to do this, from like a long time ago, but she never told me why! But I do it, every time. Even physical activity, some people will say, you don’t sleep enough. Why are you walking? You know? Things like that. You should just let your husband drive you, things like that. "Why are you so busy? You have to rest." You know? "You're pregnant.” (BMI 30 kg/m*^*2*^*; Active)*

There appeared to be conflicting ideas regarding activity during pregnancy. Whilst most women reported that their families were encouraging them to rest and take it easy, many women also reported on being warned of the dangers of too much rest. Many women alluded to the concept of being lazy during pregnancy, and believed this excessive rest was associated with adverse delivery outcomes:

*“There’s this myth that if you sleep a lot during the day the baby gets lazy, and when it’s nearer to giving birth, the baby won’t know when it’s time…you might get issues with the baby still sitting and the baby doesn’t want to come.” (BMI 26.8 kg/m*^*2*^*; Active)*

## Discussion

The purpose of this study was to qualitatively assess the beliefs, and elicit views and experiences, regarding physical activity during pregnancy in black South African women. In the current study, pregnant women described many positive beliefs about physical activity, which aligns with previous research [27, 29]. Although some women mentioned walking as a preferred activity, many defined physical activity as activities of daily living. This is common in studies of women of low SES, who often consider exercise as synonymous with work, housework or childcare [7, 30]. Indeed, despite their beliefs in the benefits of physical activity, the majority (54 %) of women in this, and other studies [11, 31], remain inactive during the prenatal period.

The discord between favourable attitudes towards physical activity, and the actual behaviour, may be explained by the factors which influence pregnant womens’ perception of control [32]. Certain pregnancy-specific physical limitations appear to be a common intrinsic barrier to perceived volitional control of being active [33]. Since all the women in the current study were in their third trimester, it is unsurprising that the issues of tiredness and size would become a barrier. Other research has reported similar physical limitations [7, 20, 27] and Hausenblas et al. [14] propose that it is these psychological and biological consequences of pregnancy that may contribute to the declining physical activity levels during this period. Interestingly, there is much research to support the role of physical activity in reducing many of these pregnancy discomforts [34], although this was not mentioned by the women in the current study. It appears that these barriers influenced both the women’s self-efficacy (it becomes harder to do the behaviour), and the controllability (‘*I listen to the baby*’). It has become evident that this combination reduces many women’s perceived behavioural control and their subsequent activity levels [24], whilst other women are determined not to let it hinder them (‘*you are not sick you are just pregnant’).*

These physical barriers were also combined with extrinsic obstacles such as lack of time, finances and adequate information, all of which are common in low socioeconomic pregnant women [25, 35]. Indeed, with the majority of the women in the current study being single, and a quarter being unemployed, many of the women may not have the perceived social, or financial support, to engage in physical activity outside of their daily routines [30]. Therefore, strategies to support these women to gain a sense of control through education and counselling may be a possible method of empowering and motivating women to embark on an active lifestyle despite these perceived obstacles.

Group physical activity may provide a powerful motivator for changing behaviours and adopting a more active lifestyle during pregnancy. Kieffer et al. [7] suggested centre-based group exercises, delivered in the context of addressing safety and developing knowledge on lifestyle behaviours, for pregnant women. This appears to be one of the most commonly suggested interventions [27] and social networks, as well as group physical activity classes, was strongly supported in this study. Interventions such as this may be a cost effective way of increasing physical activity levels, as well as a platform for providing the much needed education and information that was described as a barrier for many of these women [36].

Healthcare providers are potentially valuable motivators for physical activity [33]. However, the reported lack of accurate information, and perception of inadequate care, at the antenatal clinics diminished their influence toward improving physical activity levels. Although it may be argued that the limited healthcare resources are aimed at more eminent communicable diseases such as HIV, the overwhelming rise in non-communicable disease in South Africa warrants prioritisation within this area [37]. In fact, within the current health transition that South Africa is undergoing, non-communicable diseases are estimated to exceed HIV/AIDS as a cause for mortality [37], emphasising the importance of addressing lifestyle issues at a primary health care level. Other studies have reported a similar lack of clear, understandable advice [38], which may further reduce self-efficacy and behaviour change [25, 39]. On the other hand, our study supported the findings that increasing awareness could in fact be a facilitator for behaviour change, as women cited that they would more likely be active if they received information from their healthcare provider. A previous study by the author showed that as little as 19 % of South African medical practitioners provided information regarding physical activity during the prenatal visit [40]. Since pregnant women visit their healthcare provider on a regular basis, this may be an ideal opportunity to provide counselling and support that could improve physical activity levels [27].

This lack of guidance from health professionals prompted most women to turn to the media, or their family and friends, for advice, all of which are common sources of information during pregnancy [41, 42]. A study by Clarke et al. [42] found that less than 20 % of women’s advice was received directly from healthcare professionals. Emotional support, and a sense of accountability towards being active, has been shown in previous studies to be interpersonal facilitators of physical activity [29]. However, in the current study, many women did not have the social support network that encouraged physical activity. In fact, family advice seems to more likely discourage women from being active during pregnancy as many women, in this and previous studies, are told to “take it easy” [33, 42]. Indeed, advice to not overexert oneself during pregnancy was also echoed in some of the reported cultural beliefs in the current study. However, other cultural beliefs warned of the adverse effects of being “too lazy” or resting excessively during pregnancy. Therefore, there appears to be a contradictory tension between the social norms of taking it easy, and too much rest. Without clear guidance from healthcare professionals, this will likely lead to confusion and uncertainty surrounding an active lifestyle.

The lack of social role models, coupled with the absence of adequate information, has been shown to be a predictor of decreased physical activity levels during pregnancy [30]. Therefore, strategies to promote physical activity behaviours in these communities that include family and friends may have a more influential role than strategies directed at the pregnant women alone. Added to this, previous authors [13] have suggested a holistic approach to prenatal care to improve physical activity compliance. In a previous South African study, Muzigaba et al. [43] suggested using instructional resources such as posters, brochures and DVDs to promote healthy behaviours within the clinic setting. Added to provision of comprehensive education, we recommend incorporating physical education workers to provide exercise programmes, and social support within the community.

### Limitations

This study reached saturation after 13 interviews, and therefore utilized a small, homogenous sample of urbanised black South African women, and may not be representative of other races, cultures or communities. Therefore, the findings from this study should be applied with caution to other contexts and sub-cultures. However, very little research has been done in black African women, and our study adds to the theory-based research upon which intervention protocols for pregnant women can be based.

Furthermore, the interviews were conducted in English, and although all women could speak English, there may have been a small percentage that could not have been able to fully express themselves. Despite these limitations, the current study provided rich data that reflects unique cultural beliefs and environmental context.

## Conclusions

Since pregnancy is a crucial time for prevention of disease in both the mother and her offspring [2], it is essential that stakeholders prioritise this time for implementing physical activity interventions. The findings of this study highlight the need for proper physical activity education that is culturally sensitive, as well as social support for physical activity behaviour within this community. Incorporating the immediate and wider social community into interventions may help to diminish perceived barriers whilst at the same time influencing key role players in the woman’s support system. Designing interventions that are contextually and culturally specific, may promote physical activity behavioural change in not only the pregnant population, but at a community level as well.

## Abbreviations

BMI, body mass index; CHBH, Chris Hani Baragwaneth hospital; DPHRU, developmental pathways for health research unit; G, gravidity; GDM, gestational diabetes mellitus; GPAQ, global physical activity questionnaire; LMICs, low-to-middle income countries; MRC, medical research council; PBC, perceived control beliefs; SES, socioeconomic status; TPB, theory of planned behaviour
